# Structural Characterization of an N-Acetyl Sugar Amidotransferase Involved in the Lipopolysaccharide Biosynthesis in Bacteria

**DOI:** 10.3390/ijms242015491

**Published:** 2023-10-23

**Authors:** Jiajia Gao, Wenwen Xu, Tianqi Liu, Wenjie Sun, Na Wang, Jinming Ma, Honghua Ge

**Affiliations:** 1 Information Materials and Intelligent Sensing Laboratory of Anhui Province, Institutes of Material Science and Information Technology, Anhui University, Hefei 230601, China; 23748@ahu.edu.cn (J.G.); 21043@ahu.edu.cn (N.W.); 2 School of Resources and Environmental Engineering, Anhui University, Hefei 230601, China

**Keywords:** *Legionella pneumophila*, LPS biosynthesis, N-acetyl sugar amidotransferase, Rossmann-like fold, PP-loop

## Abstract

N-acetyl sugar amidotransferase (NASAT) is involved in the lipopolysaccharide (LPS) biosynthesis pathway that catalyzes the formation of the acetamido moiety (sugar-NC(=NH)CH3) on the O-chain. So far, little is known about its structural and functional properties. Here, we report the crystal structure of an N-acetyl sugar amidotransferase from *Legionella pneumophila* (LpNASAT) at 2.33 Å resolution. LpNASAT folds into a compact basin-shaped architecture with an unusually wide and open putative substrate-binding pocket and a conserved zinc ion-binding tetracysteine motif. The pocket contains a Rossmann-like fold with a PP-loop, suggesting that the NASAT-catalyzed amidotransfer reaction probably requires the conversion of ATP to AMP and PPi. Our data provide structural insights into the NASAT family of proteins, and allow us to possibly identify its functionally important regions.

## 1. Introduction

Lipopolysaccharide (LPS) is a complex glycolipid which is an essential component of the cell envelope in most Gram-negative bacteria [1]. It plays an important role in the stability of the bacterial outer membrane. Due to its physicochemical properties, LPS can inhibit the passage of small, hydrophobic molecules through the phospholipid bilayer, endowing bacteria with an innate resistance to external agents (e.g., antibiotics and detergents) [2]. As an endotoxin, LPS also plays a crucial role in bacteria–host interactions by modulating the host immune system’s responses [3].

LPS consists of three components that are covalently linked to each other in descending order: lipid A, a core oligosaccharide, and a long polysaccharide (O-chain). Unlike the other components, O-chains are known as the most variable structures in LPS molecules and confer resistance to complement-mediated killing [4]. They are composed of a variable number of repeating oligosaccharide units that extend outside the bacteria.

**Figure 1:**
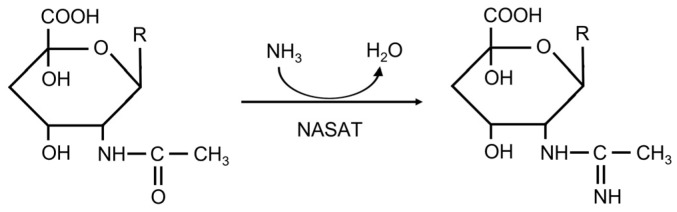
Amidotransfer reaction catalyzed by NASAT.

During O-chain biosynthesis, N-acetyl sugar amidotransferase (NASAT) has been implicated in the formation of the acetamido moiety (sugar-NC(=NH)CH3). The reaction involves the ligation of ammonia with a sugar N-acetyl group, displacing water (Figure 1). The NASAT family belongs to the AANH_like (Adenine nucleotide alpha hydrolases-like) superfamily [5], whose domain forms an alpha/beta/alpha fold for Adenosine nucleotide binding [6]. Members of the NASAT family carry a conserved PP-loop (SGGXDS, where X represents any residue), indicating that they may have ATP PPase activity. In addition, this family is characterized by a conserved tetracysteine motif (CXXCX_n_[GN]XCXXC, where X represents any residue). The site was supposed to bind a metal atom, whose nature was, however, unknown.

According to previous studies, WbpG, a member of NASAT family in *Pseudomonas aeruginosa O5*, is responsible for forming the C-3 acetiminido group on the first sugar residue of the O-unit to produce DManNAc3NAmA [7,8]. WbuX in *E. coli O145* is a homolog of WbpG, which is involved in the amination of L-FucNAc for the synthesis of L-FucNAm [9]. However, while NASAT is indispensable for generating bacterial LPS specificity, surprisingly little is known about its structural and functional properties.

We herein report for the first time the crystal structure of NASAT from *Legionella pneumophila* (LpNASAT, Uniprot ID: A0A3A6V1D8_LEGPN) at 2.33 Å resolution. The elucidated structure provides detailed structural information pertaining to the NASAT family, and allows us to possibly identify its functionally important regions.

## 2. Results and Discussion

### 2.1. Overall Structure

**Figure 2:**
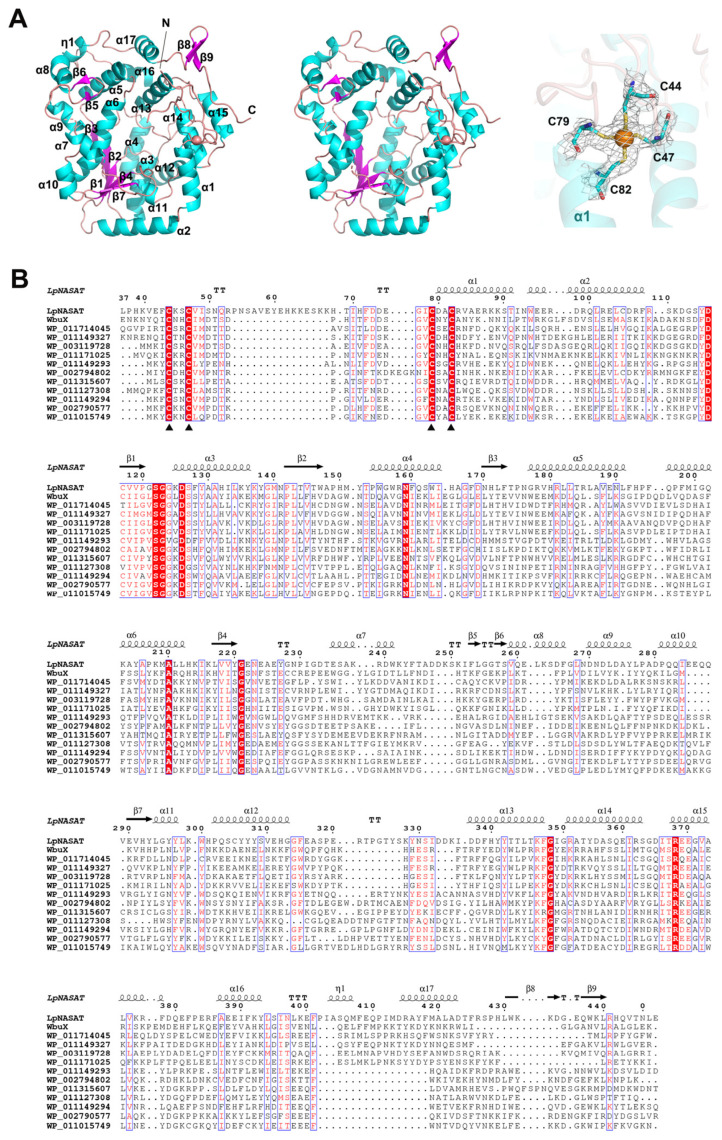
Crystal structure of LpNASAT. (**A**) Stereo view of cartoon representation of LpNASAT structure. The N and C termini and secondary structure elements are labeled. The zinc ion is drawn as orange sphere. Electron density map contoured at 1.5 σ for Zn-binding tetracysteine motif. (**B**) Structure-based Sequence alignment of LpNASAT and its homologs. The sequence of LpNASAT from *L. pneumophila* (PDB code 8WEX; present study) was aligned with the sequences of WbuX from *E. Coli*, WbpG (WP_003119728) from *P. aeruginosa PAO1*, WP_011714045 from *M. marinus*, WP_011149327 from *V. vulnificus*, WP_011171025 from *M. maripaludis*, WP_011149293 from *V. vulnificus*, WP_002794802 from *Campylobacter*, WP_011315607 from *N. winogradskyi*, WP_011127308 from *P. marenigrum*, WP_011149294 from *V. vulnificus*, WP_002790577 from *Campylobacter*, and WP_011015749 from *F. nucleatum*. The alignment was performed using Clustal Omega [15] and ESPript [16]. α-helices, β-strands and 3_10_-helices are denoted by Greek characters α, β and η, respectively. Identical residues are shown in white with a red background and conservative changes in red with a white background. Zn-binding cysteines are indicated by black triangles.

We determined the crystal structure of LpNASAT, and refined it to an R factor (R_free_) of 20.6% (24.9%) (Table 1). Most residues are modelled in two chains. A few regions, including long N-terminal residues, several gaps, and 11 C-terminal residues were not modeled due to the poor electron density map in their respective regions, indicating the high flexibility of these areas. The structures of chains A and B are nearly identical with a RMSD of 0.137 Å for 327 common Cα atoms. The protein contains 18 helices and nine strands (Figure 2A,B). A five-stranded parallel β-sheet (β3β2β1β4β7) in the core (lower part) of the molecule was sandwiched by two arrays of α-helices (α3α4α6α10α11), which is a typical nucleotide-binding Rossmann-like fold [10]. The remainder is packed around the Rossmann-like fold to complete a compact architecture, resulting in a basin shape and creating a big pocket.

On the edge of the pocket, there is a conserved tetracysteine motif (Cys44, Cys47, Cys79 and Cys82) possibly indicative of a metal binding site. Not surprisingly, the omit map calculated using protein sequence alone reveals a significant positive density close to the tetracysteine motif. To confirm the identity of the metal, we conducted inductively coupled plasma mass spectroscopy (ICP-MS) experiments on highly purified LpNASAT, and the results showed that zinc appears to be the candidate among the transition metals under investigation (Figure 2A and Figure S1).

Based on structural comparison with experimentally solved data performed at the Dali server, the closest structural homologs of LpNASAT include a Sulfur transferase from Pyrococcus horikoshii OT3 (PDB code 5mko; Z score: 10.4; rmsd: 3.8; sequence identity: 14%) [11], 2-thiouridine synthetase TtuA from Thermus thermophilus HB27 (PDB code 5b4e; Z score: 10.1; rmsd: 4.1; sequence identity: 13%) [12], Nicotinamide mononucleotide synthetase from Francisella tularensis (FtNadE) (PDB code 3fiu; Z score: 9.6; rmsd: 4.0; sequence identity: 9%) [13] and sulfur transferase LarE from Lactiplantibacillus plantarum (PDB code 6b2m; Z score: 9.3; rmsd: 4.0; sequence identity: 10%) [14]. These proteins all contain a Rossmann-like fold. Superposition of the structures showed that despite of the similarity in the Rossmann-like fold region, there are notable differences in overall architecture (Figure S2).

### 2.2. LpNASAT Crystallizes as a Dimer

**Figure 3:**
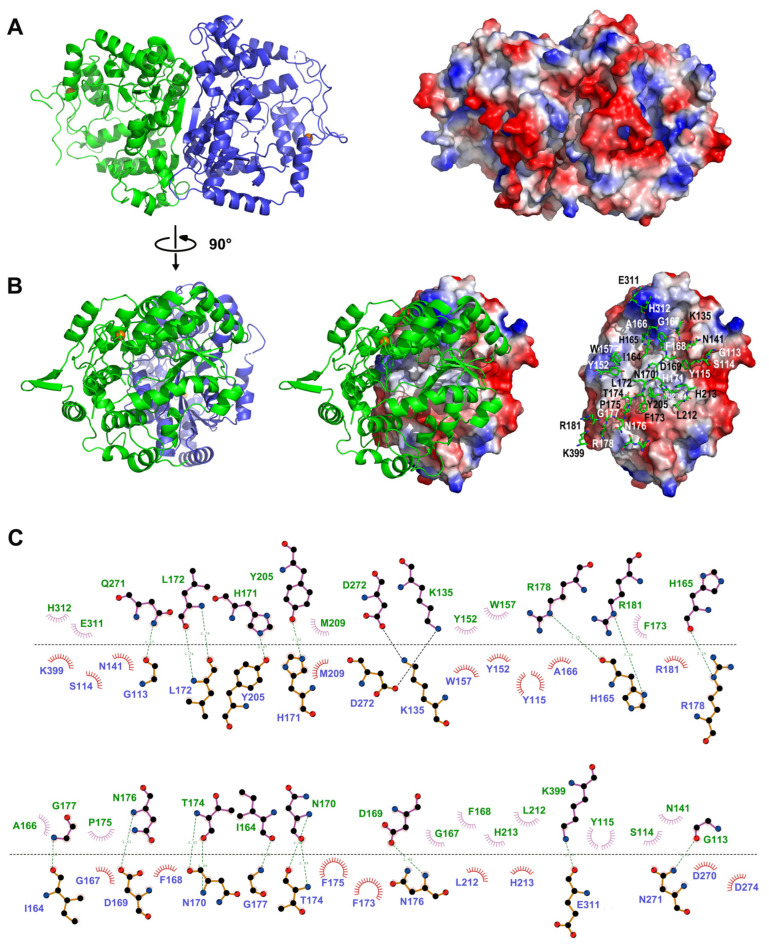
LpNASAT crystallizes as a dimer. (**A**) Two LpNASAT molecules in the asymmetric unit form a dimer. The left panel shows the ribbon representation of the dimer organization of LpNASAT with each monomer colored separately. The right panel shows electrostatic potential surface plots of LpNASAT dimer. The structure is represented on the surface at the same orientation as in the left panel. Surface electrostatic potential map was generated by PyMol [18], with positive and negative regions in blue and red, respectively. (**B**) Closed-up view of the interactions of two neighboring monomers. The view differs by a 90° rotation along the vertical axis. The relevant residues in one monomer are labeled and shown in stick form. (**C**) Ligplot diagram illustrating the molecular interactions in a LpNASAT dimer. Hydrogen bonding and hydrophobic interactions were illustrated using Ligplot [19].

Two LpNASAT molecules in the asymmetric unit seem to form a dimer (Figure 3A,B). PISA (Proteins, Interfaces, Structures and Assemblies) program [17] analysis of protein the interfaces suggests that the dimer is likely to be stable in solution with a favorable interaction energy (ΔiG −3.9 kcal/mol), consistent with the results of size exclusion chromatography (Figure S3). The dimer interface covers about 1632 Å^2^, corresponding to 8.7% of the total accessible surface area of one monomer (about 18,941 Å^2^).

According to the analysis of LIGPLOT [19], the dimer interfaces reveal an extensive network of interactions that involves hydrogen bonds and hydrophobic contacts (Figure 3B,C). Residues Gly113, Lys135, Ile164, His165, Asp169, Asn170, His171, Leu172, Thr174, Asn176, Gly177, Arg178, Arg181, Tyr205, Gln271, Asp272, Glu311 and Lys399 involve in direct hydrogen bonds. Besides, residues Ser114, Tyr115, Asn141, Tyr152, Trp157, Ala166, Gly167, Phe168, Phe173, Pro175, Met209, Leu212, His213, Asp270, Asp274, Glu311 and His312 contribute to a large number of hydrophobic interactions. There is also a small number of water-molecule-mediated hydrogen bonding interactions at the dimerization interface. These interactions together stabilize the structure of the dimer. However, the vast majority of these residues are not conserved among the NASAT family members (Figure 2B and Figure S4), suggesting that proteins in this family may not necessarily require dimerization to function.

### 2.3. Possible Functional Regions

**Figure 4:**
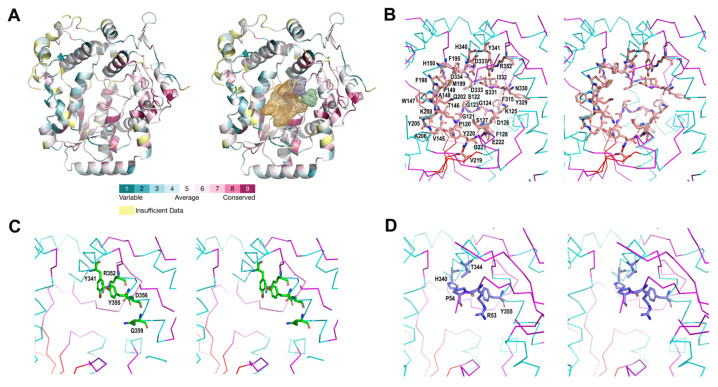
Closed-up view of the putative substrate-binding pocket. (**A**) Location of the possible binding sites of LpNASAT obtained from the FTSite server. The yellow, blue and green mesh represent different binding spaces. The conservation pattern was obtained using the Consurf server [21]. The correspondence between conservation and color is labeled from the most variable (turquoise) through intermediately conserved positions (white) to the most conserved (burgundy). (**B**–**D**) Close-up view of the binding sites. The residues within 5 Å of the binding sites are labeled and shown in stick form.

The binding site of LpNASAT was predicted using the FTSite binding site predicting tool [20]. The yellow, blue and green mesh in the big pocket represent the predicted binding sites (Figure 4A) and the residues within 5 Å of the binding sites are shown in Figure 4B–D. According to the conservation pattern analysis (Figure 2B and Figure 4A), the side of the putative substrate-binding pocket near the tetracysteine motif contains conserved negatively charged amino acids that are likely essential for catalyzing the amidotransfer reaction. At the other side of the pocket near α6, there are a number of positively charged residues which are highly variable in homologs from other bacterial species, suggesting possible major differences in substrate structure.

**Figure 5:**
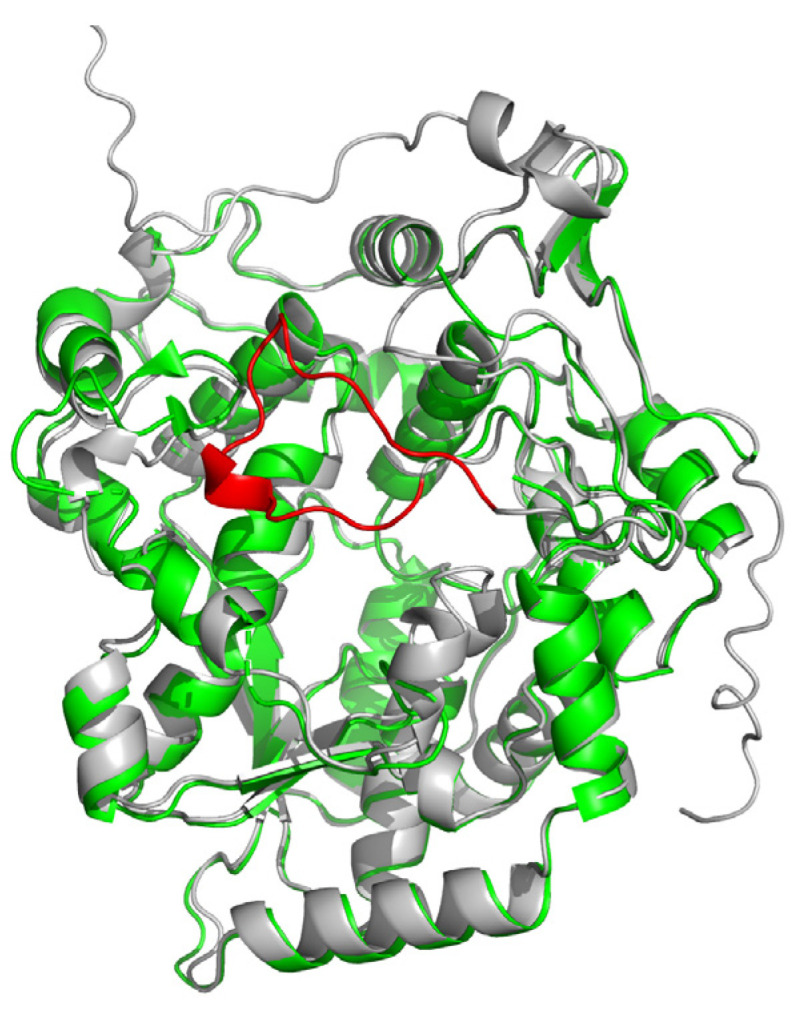
Superposition of the crystal structure (PDB code 8WEX, green) and AlphaFold-predicted structure (grey) of LpNASAT. The fragment (residues 56–69) is shown in red.

According to the electron density map, a fragment consisting of approximately fifteen residues (residues 55–69) is severely disordered. Based on its orientation, the fragment is probably located above the putative substrate-binding pocket. The analysis of AlphaFold [22] predicted that the structure of LpNASAT supports this hypothesis (Figure 5). Therefore, we hypothesize that this region maybe be involved in the regulation of substrate binding and product release. Interestingly, this fragment is missing in other NASAT family members. This suggests that there may be a difference in the catalytic mechanism between LpNASAT and other members.

**Figure 6:**
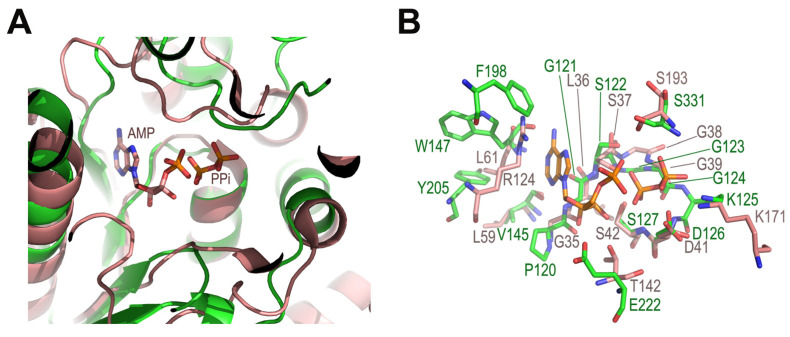
Superposition of the ligand-binding site of FtNadE and LpNASAT. (**A**) The PP-loop of FtNadE in complex with the products of ATP hydrolysis (PDB code 3fiu, orange) and LpNASAT (PDB code 8WEX, green). (**B**) Close-up view of the nucleotide-binding site. The amino acid residues, which are involved in FtNadE-AMP-PPi binding, are shown in orange. Residues of LpNASAT at positions equivalent to those of FtNadE are shown in green.

At the bottom of the putative substrate-binding pocket, LpNASAT harbors a highly conserved PP-loop motif (122-SGGKDS-127) on a region between β1 and α3. The PP-loop motif is the signature of the ATP pyrophosphatase domains [23], which catalyzes the cleavage of the bond between α and β phosphates of ATP to form AMP and PPi. Due to the amino groups of the loop and dipole of helix α3, the PP-loop forms a large anionic space, which may contribute to the binding of nucleotide phosphate groups. In the electron density map of the crystal, we observed a distinct density feature close to the PP-loop motif (Figure S5). When the LpNASAT and FtNadE (PDB code 3fiu, AMP-PPi-bound structure) [13] structures are superimposed, the PPi moiety in FtNadE is equivalent to the position of the observed density in LpNASAT. The residues, which are involved in specific hydrogen bonds and van der Waals contacts to the AMP and PPi in FtNadE, are conserved or have similar physicochemical properties to the corresponding residues in LpNASAT and its homologs (Figure 6). Based on these observations, we speculated that the amidotransfer reaction catalyzed by NASAT family members is likely to utilize the energy provided by the hydrolysis of ATP to AMP.

## 3. Materials and Methods

### 3.1. Expression and Purification of Recombinant LpNASAT

The recombinant construct of LpNASAT cloned with an N-terminal 6xHis-tag into the bacterial expression vector p28 derived from pET28a by deleting the sequence AGCAGCGGCCTGGTGCCGCGCGGCAGC between the NcoI and NdeI restriction sites. This recombinant plasmid was transformed into Escherichia coli strain Rosetta. Cells were grown at 37 °C in Luria–Bertani medium containing 100 μg/mL Kanamycin. Expression of the recombinant LpNASAT was induced at an OD600 of 0.8–1.0 by adding 0.2 mM isopropyl -β-d-1-thiogalactopyranoside (IPTG) followed by incubation at 16 °C for 20 h. The cells were harvested by centrifugation at 4 °C, 6000× *g* for 10 min. Harvested cells were re-suspended in 20 mM Tris-HCl buffer pH 8.0 containing 400 mM NaCl and lysed by using French Press. The lysate was clarified by centrifugation at 15,000× *g* for 30 min at 4 °C. The soluble fraction was loaded onto a Chelating Sepharose Fast Flow (GE Healthcare, Chicago, IL, USA) pre-equilibrated with 20 mM Tris pH 8.0, 400 mM NaCl. The resin was washed with 20 mM Tris-HCl pH 8.0, 50 mM imidazole, 400 mM NaCl, and the target protein was eluted with 20 mM Tris-HCl pH 8.0, 250 mM imidazole, 400 mM NaCl. The eluate was further purified using HiLoad 16/60 Superdex 200 (GE Healthcare). The eluted recombinant LpNASAT was concentrated and stored in 20 mM Tris-HCl buffer (pH 8.0) containing 400 mM NaCl. The protein concentration was determined using the Bradford method (Bio-Rad Protein Assay, Hercules, CA, USA), using bovine serum albumin as standard.

### 3.2. Crystallization, Data Collection and Structure Determination

Crystals of LpNASAT were produced by the hanging-drop vapor-diffusion method. An amount of 2 μL of protein solution (10 mg/mL) was mixed with 2 μL of precipitant solution (20% (*w*/*v*) PEG 3350, 0.2 M ammonium acetate) and incubated at 14 °C. The crystals were harvested using cryoloops and immersed briefly in a cryoprotectant solution consisting of 80% (*v*/*v*) reservoir solution and 20% (*v*/*v*) glycerol. The crystals were subsequently flash-frozen and stored in liquid nitrogen for further data collection. The best data were collected in beamline BL17U1 of the SSRF (Shanghai Synchrotron Radiation Facility) and processed by AutoPX [24]. Using a RoseTTAFold [25] predicted model as a search coordinate, the LpNASAT structure was determined by molecular replacement with the Phaser program [26]. The model was completed by iterative manual building in Coot [27] and refined with REFMAC5 [28] and PHENIX [29]. The quality of the final refined model was evaluated using MolProbity [30]. The statistics of data collection and refinement are summarized in Table 1.

The DALI [31] was used for structure similarity search. Amino acid sequences were aligned by Clustal Omega [15], and the figure of structure-based sequence alignment was generated using ESPript [16]. All other structural figures were prepared with PyMOL [18].

### 3.3. Inductively Coupled Plasma Mass Spectroscopy (ICP-MS)

The metal contents in the LpNASAT sample were measured using Thermo Scientific iCAP Q ICP-MS. The purified protein and buffer solution were heated at 65 °C in 2 M HNO3 for 20 min, kept at room temperature overnight, and centrifuged at 14,000× *g* for 20 min. The metal concentrations of the common transition metals (Cu, Ge, Mg, Mn, Ni, Fe, Zn, Co and Ru) in the supernatant were analyzed by ICP-MS. All samples were measured in three replicates.

### 3.4. Size-Exclusion Chromatography

The molecular mass and oligomeric state of LpNASAT were characterized on an AKTA FPLC (GE Healthcare) using a Superdex™ 200 Increase column 10/300 GL (Cytiva, Lot 10323365, Marlborough, MA, USA). The column was equilibrated with 20 mM Tris-HCl, pH 8.0, 400 mM NaCl and run at 0.7 mL/min at 16 °C. A calibration curve for molecular size estimation was generated by loading thyroglobulin (670 kDa), gamma globulin (158 kDa), ovalbumin (44 kDa), myoglobin (17 kDa), and vitamin B12 (1.35 kDa) on this analytical column and eluting was conducted under the same conditions.

### 3.5. ConSurf Analysis of Evolutionary Conservation

The evolutionary conservation profile of LpNASAT was estimated using the ConSurf tool [21]. The homolog search of the LpNASAT sequence was performed against the UNIREF-90 database with 1 HMMER iteration, an E-value cutoff of 0.0001, minimal % ID of 35% for homologs and maximal % ID of 95% between sequences. A total of 150 homologous sequences were retrieved and multiply aligned using MAFFT. The calculation of conservation scores of each residue was performed using an empirical Bayesian method.

## Figures and Tables

**Table 1:** Data collection and refinement statistics.

	LpNASAT
Data collection	
SSRF beamline	BL17U
Wavelength (Å)	0.97914
Space group	*P*32_1_2
Molecules/ASU	2
Cell parameters	
a/b/c (Å) α/β/γ	74.72/74.72/319.68 90/90/120
Resolution range (Å)	45.67–2.33 (2.39–2.33)
No. of unique reflections	44,179 (3210)
*R_pim_* ^1^ (%)	4.6 (28.6)
Average I/σ(I)	19.8 (2.7)
CC1/2	0.998 (0.602)
Completeness (%)	100 (100)
Redundancy	17.2 (17.4)
Refinement	
PDB entry	8WEX
Resolution limits (Å)	19.99–2.33 (2.42–2.33)
No. of reflections	44,010 (4224)
R factor ^2^ (%)	20.6 (29.7)
Free R factor ^3^ (%)	24.9 (36.5)
No. of protein atoms	6236
No. of ligands	2
No. of solvent molecules	232
rmsd ^4^ in bond lengths (Å)	0.012
rmsd in bond angles (°)	1.51
Wilson B-factor (Å^2^)	45.5
Average B-factor (Å^2^)	55.8
macromolecules	56.0
ligands	57.0
waters	49.1
Ramachandran plot (%)	
favored/disallowed	96.68/0

Values in parentheses refer to the highest resolution shell. ^1^ *R_pim_* = ∑*_hkl_* [1/(n*_hkl_* − 1)]^1/2^∑*_i_* |I*_i_*(*hkl*) − 〈I(*hkl*)〉|/∑*_hkl_* ∑*_i_*I*_i_*(*hkl*), where n*_hkl_* is the number of observations of reflection *hkl*. ^2^ R-factor = ∑*h* | |*F_obs_*| − |*F_calc_*| |/∑|*F_obs_*|, where |*F_obs_*| and |*F_calc_*| are the observed and calculated structure factor amplitudes, respectively. Summation includes all reflections used in the refinement. ^3^ Free R factor = ∑| |*F_obs_*| − |*F_calc_*| |/∑|*F_obs_*|, evaluated for a randomly chosen subset of 5% of the diffraction data not included in the refinement. ^4^ Root-mean square-deviation from ideal values.

## Data Availability

The coordinates and structure factors have been deposited in the Protein Data Bank under the accession code 8WEX.

## Supplementary Materials

The supporting information can be downloaded at https://www.mdpi.com/article/10.3390/ijms242015491/s1.

## References

[1] Zhang G., Meredith T.C., Kahne D. (2013). On the essentiality of lipopolysaccharide to Gram-negative bacteria. Curr. Opin. Microbiol..

[2] Nikaido H. (2003). Molecular basis of bacterial outer membrane permeability revisited. Microbiol. Mol. Biol. Rev..

[3] Bertani B., Ruiz N. (2018). Function and Biogenesis of Lipopolysaccharides. EcoSal Plus.

[4] Dasgupta T., de Kievit T.R., Masoud H., Altman E., Richards J.C., Sadovskaya I., Speert D.P., Lam J.S. (1994). Characterization of lipopolysaccharide-deficient mutants of *Pseudomonas aeruginosa* derived from serotypes O3, O5, and O6. Infect. Immun..

[5] Lu S., Wang J., Chitsaz F., Derbyshire M.K., Geer R.C., Gonzales N.R., Gwadz M., Hurwitz D.I., Marchler G.H., Song J.S. (2020). CDD/SPARCLE: The conserved domain database in 2020. Nucleic Acids Res..

[6] Aravind L., Anantharaman V., Koonin E.V. (2002). Monophyly of class I aminoacyl tRNA synthetase, USPA, ETFP, photolyase, and PP-ATPase nucleotide-binding domains: Implications for protein evolution in the RNA. Proteins.

[7] King J.D., Kocincova D., Westman E.L., Lam J.S. (2009). Review: Lipopolysaccharide biosynthesis in *Pseudomonas aeruginosa*. Innate Immun..

[8] Rocchetta H.L., Burrows L.L., Lam J.S. (1999). Genetics of O-antigen biosynthesis in *Pseudomonas aeruginosa*. Microbiol. Mol. Biol. Rev..

[9] Feng L., Senchenkova S.N., Tao J., Shashkov A.S., Liu B., Shevelev S.D., Reeves P.R., Xu J., Knirel Y.A., Wang L. (2005). Structural and genetic characterization of enterohemorrhagic *Escherichia coli* O145 O antigen and development of an O145 serogroup-specific PCR assay. J. Bacteriol..

[10] Medvedev K.E., Kinch L.N., Schaeffer R.D., Grishin N.V. (2019). Functional analysis of Rossmann-like domains reveals convergent evolution of topology and reaction pathways. PLoS Comput. Biol..

[11] Arragain S., Bimai O., Legrand P., Caillat S., Ravanat J.L., Touati N., Binet L., Atta M., Fontecave M., Golinelli-Pimpaneau B. (2017). Nonredox thiolation in tRNA occurring via sulfur activation by a [4Fe-4S] cluster. Proc. Natl. Acad. Sci. USA.

[12] Chen M., Asai S.I., Narai S., Nambu S., Omura N., Sakaguchi Y., Suzuki T., Ikeda-Saito M., Watanabe K., Yao M. (2017). Biochemical and structural characterization of oxygen-sensitive 2-thiouridine synthesis catalyzed by an iron-sulfur protein TtuA. Proc. Natl. Acad. Sci. USA.

[13] Sorci L., Martynowski D., Rodionov D.A., Eyobo Y., Zogaj X., Klose K.E., Nikolaev E.V., Magni G., Zhang H., Osterman A.L. (2009). Nicotinamide mononucleotide synthetase is the key enzyme for an alternative route of NAD biosynthesis in *Francisella tularensis*. Proc. Natl. Acad. Sci. USA.

[14] Fellner M., Rankin J.A., Desguin B., Hu J., Hausinger R.P. (2018). Analysis of the Active Site Cysteine Residue of the Sacrificial Sulfur Insertase LarE from *Lactobacillus plantarum*. Biochemistry.

[15] Sievers F., Higgins D.G. (2018). Clustal Omega for making accurate alignments of many protein sequences. Protein Sci. Publ. Protein Soc..

[16] Robert X., Gouet P. (2014). Deciphering key features in protein structures with the new ENDscript server. Nucleic Acids Res..

[17] Krissinel E., Henrick K. (2007). Inference of macromolecular assemblies from crystalline state. J. Mol. Biol..

[18] Schrodinger, LLC (2015). The PyMOL Molecular Graphics System.

[19] Laskowski R.A., Swindells M.B. (2011). LigPlot+: Multiple ligand-protein interaction diagrams for drug discovery. J. Chem. Inf. Model..

[20] Ngan C.H., Hall D.R., Zerbe B., Grove L.E., Kozakov D., Vajda S. (2012). FTSite: High accuracy detection of ligand binding sites on unbound protein structures. Bioinformatics.

[21] Ashkenazy H., Abadi S., Martz E., Chay O., Mayrose I., Pupko T., Ben-Tal N. (2016). ConSurf 2016: An improved methodology to estimate and visualize evolutionary conservation in macromolecules. Nucleic Acids Res..

[22] Jumper J., Evans R., Pritzel A., Green T., Figurnov M., Ronneberger O., Tunyasuvunakool K., Bates R., Zidek A., Potapenko A. (2021). Highly accurate protein structure prediction with AlphaFold. Nature.

[23] Fellner M., Hausinger R.P., Hu J. (2018). A structural perspective on the PP-loop ATP pyrophosphatase family. Crit. Rev. Biochem. Mol..

[24] Wang L., Yun Y., Zhu Z., Niu L. (2022). AutoPX: A new software package to process X-ray diffraction data from biomacromolecular crystals. Acta Crystallogr. D Struct. Biol..

[25] Baek M., DiMaio F., Anishchenko I., Dauparas J., Ovchinnikov S., Lee G.R., Wang J., Cong Q., Kinch L.N., Schaeffer R.D. (2021). Accurate prediction of protein structures and interactions using a three-track neural network. Science.

[26] McCoy A.J., Grosse-Kunstleve R.W., Adams P.D., Winn M.D., Storoni L.C., Read R.J. (2007). Phaser crystallographic software. J. Appl. Crystallogr..

[27] Emsley P., Lohkamp B., Scott W.G., Cowtan K. (2010). Features and development of Coot. Acta Crystallogr. D Biol. Crystallogr..

[28] Murshudov G.N., Skubak P., Lebedev A.A., Pannu N.S., Steiner R.A., Nicholls R.A., Winn M.D., Long F., Vagin A.A. (2011). REFMAC5 for the refinement of macromolecular crystal structures. Acta Crystallogr. D Biol. Crystallogr..

[29] Liebschner D., Afonine P.V., Baker M.L., Bunkoczi G., Chen V.B., Croll T.I., Hintze B., Hung L.W., Jain S., McCoy A.J. (2019). Macromolecular structure determination using X-rays, neutrons and electrons: Recent developments in Phenix. Acta Crystallogr. D.

[30] Chen V.B., Arendall W.B., Headd J.J., Keedy D.A., Immormino R.M., Kapral G.J., Murray L.W., Richardson J.S., Richardson D.C. (2010). MolProbity: All-atom structure validation for macromolecular crystallography. Acta Crystallogr. D Biol. Crystallogr..

[31] Holm L., Laiho A., Toronen P., Salgado M. (2023). DALI shines a light on remote homologs: One hundred discoveries. Protein Sci. Publ. Protein Soc..

