# Supplementary material for: Structural Characterization of an N-Acetyl Sugar Amidotransferase Involved in the Lipopolysaccharide Biosynthesis in Bacteria
Source: Int J Mol Sci. 2023 Oct 23;24(20):15491. doi: 10.3390/ijms242015491 (PMC10607449; doi:10.3390/ijms242015491)
Supplement: Supplementary file 1 [file ijms-24-15491-s001.zip › ijms-2652149-supplementary.pdf]

Supplementary figures

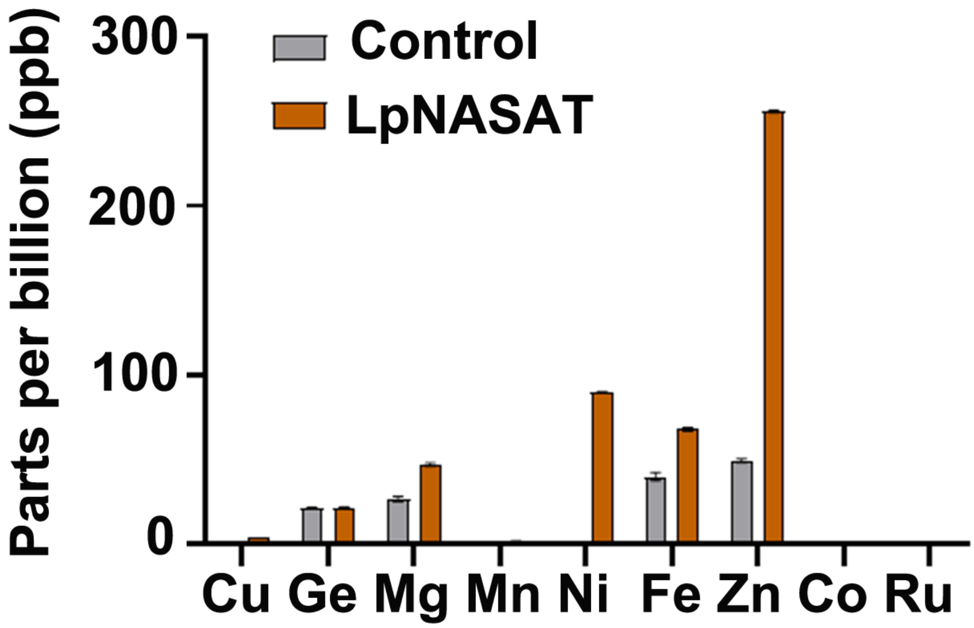

Figure S1. Metal content analysis of the purified LpNASAT by ICP-MS. The contents of nine common metals were determined in highly purified LpNASAT. For comparison, the metal contents of a control sample buffer (treated in the same way as the protein sample) were measured in parallel. All samples were measured in three replicates.

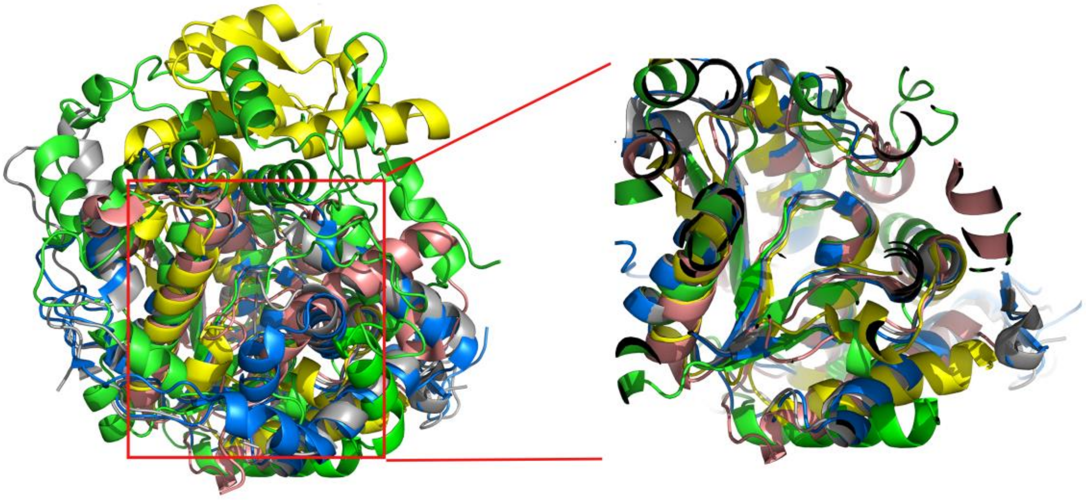

Figure S2. Superposition of the structures of LpNASAT (PDB code 8WEX, green), FtNadE (PDB code 3fiu, orange), sulfur transferase LarE from *L. plantarum* (PDB code 6b2m, yellow), Sulfur transferase from *P. horikoshii* OT3 (PDB code 5mko, blue) and Sulfur Transferase TtuA from *T. thermophilus* HB27 (PDB code 5b4e, Grey).

A

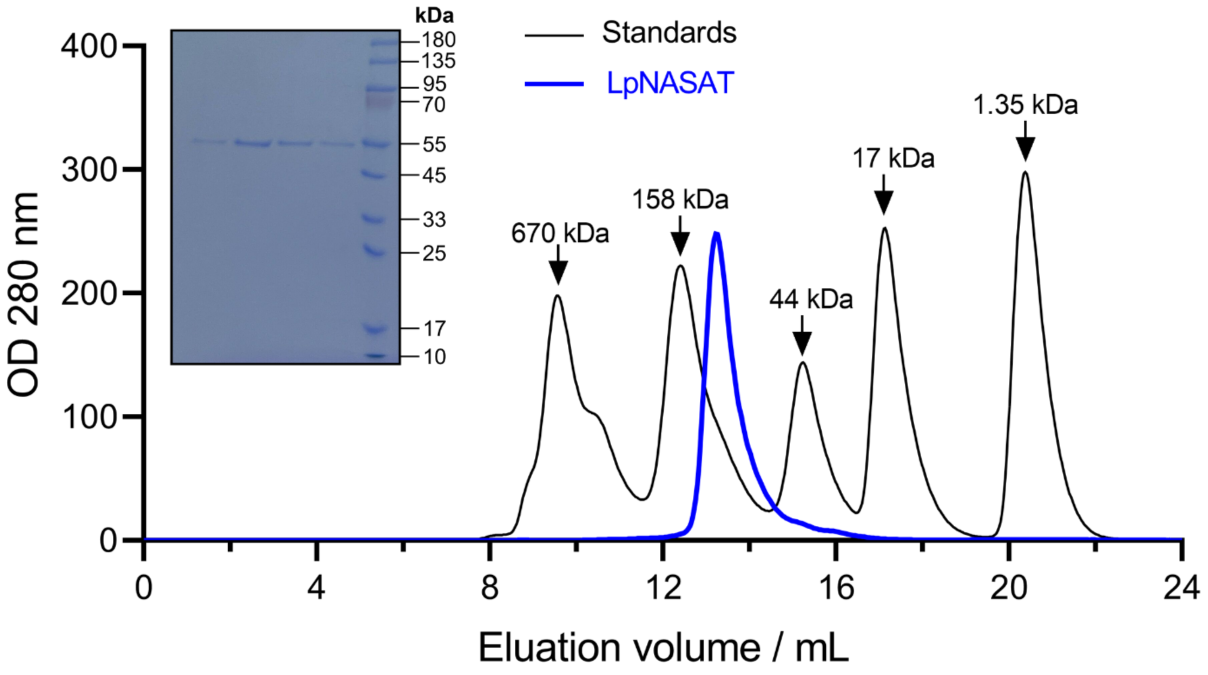

B

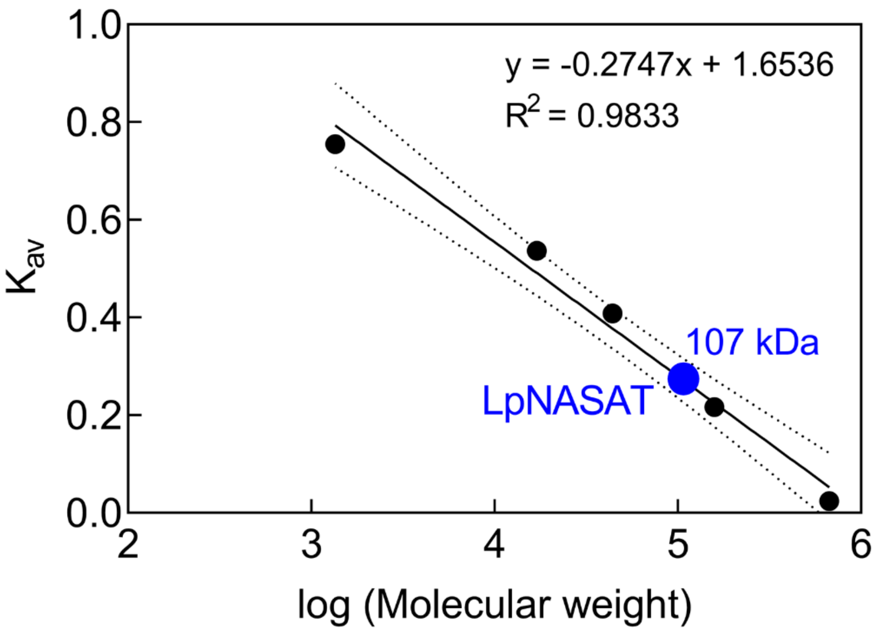

Figure S3. The results of size-exclusion chromatography (SEC) analysis. (A) The recombinant LpNASAT eluted as a single peak (blue line) between gamma globulin (158kDa) and ovalbumin (44kDa). The SEC-purified LpNASAT runs as an ~50 kDa protein band on 12 % SDS–PAGE. (B) The calibration curve with *x* and *y* axes representing the log molecular weight and average *K* (K_{av}), respectively, was used for molecular-weight estimation of LpNASAT. Thyroglobulin (670kDa), gamma globulin (158kDa), ovalbumin (44kDa), myoglobin (17kDa) and vitamin B12 (13.5kDa) was used for calibration. The estimated molecular weight of LpNASAT is approximately 107 kDa.

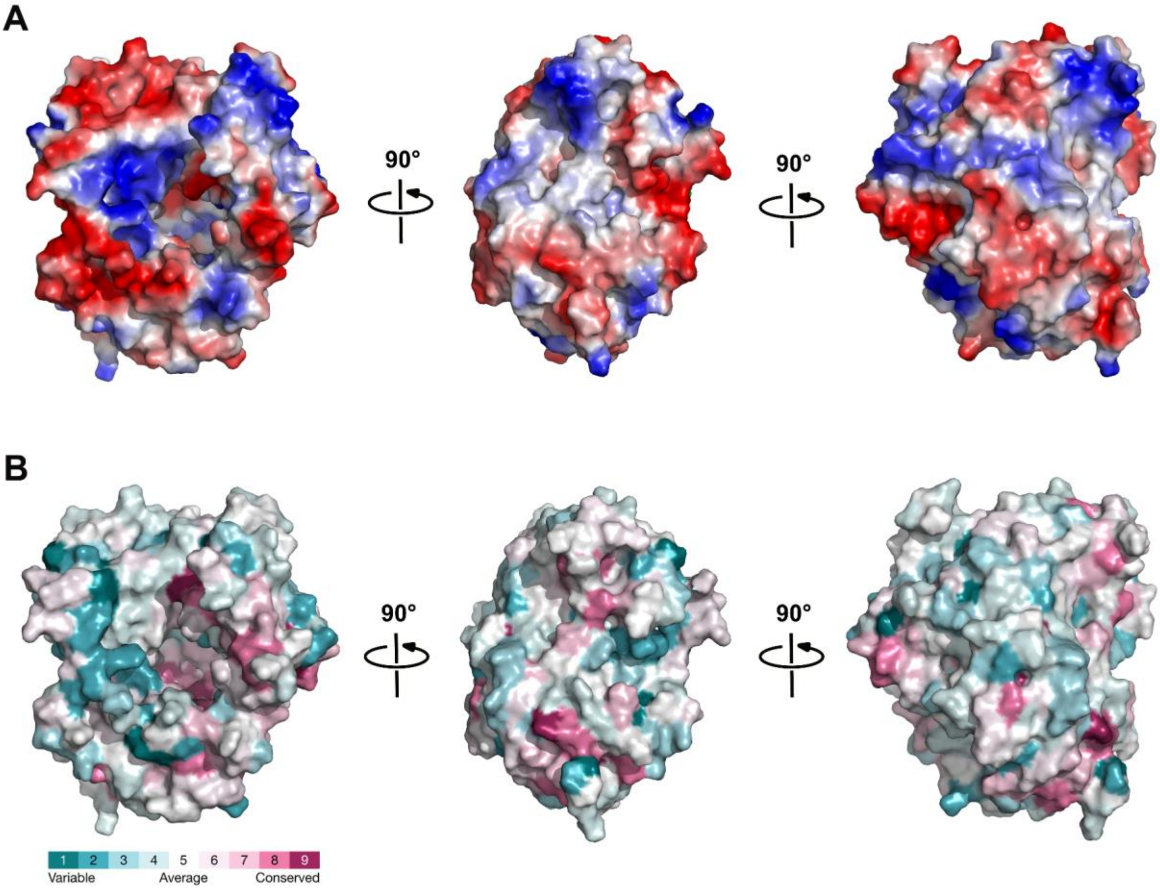

Figure S4. Surface properties of LpNASAT. (A) Electrostatic potential surface plots of LpNASAT. Surface electrostatic potential map was generated by PyMol [18], with positive and negative regions in blue and red, respectively. (B) The conservation pattern of LpNASAT. The surface representation is colored from the least conserved (turquoise) through intermediately conserved (white) to the most conserved residues (burgundy). The first image of each series (from left to right) is in the same orientation as Figure 2A.

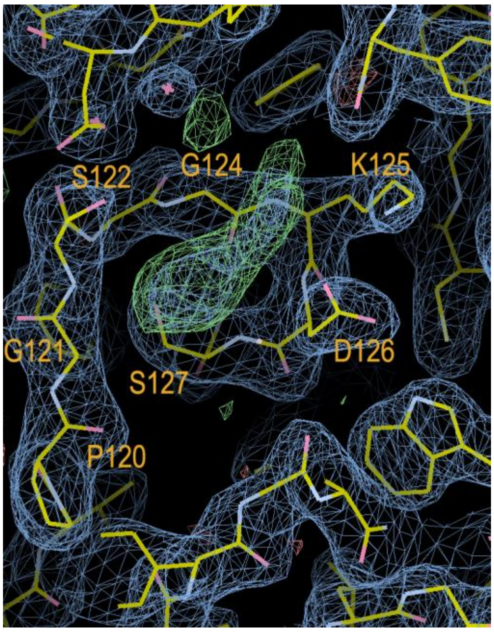

Figure S5. The electron density map of the PP-loop motif. The blue meshes ($\sigma = 1.0$) and the green meshes ($\sigma = 3.0$) show the 2Fo-Fc map and the Fo-Fc omit map, respectively.
